# Cutaneous Polyarteritis Nodosa Presenting Atypically with Severe Pharyngeal Ulceration

**DOI:** 10.1155/2019/2631948

**Published:** 2019-03-25

**Authors:** Bayanne Olabi, Justin C. Mason, Ziad Farah

**Affiliations:** ^1^NHS Lothian, Department of Dermatology, Lauriston Building, Lauriston Place, Edinburgh EH3 9EN, UK; ^2^Rheumatology Section, Imperial College Healthcare NHS Trust, Hammersmith Hospital, Du Cane Road, London W12 0HS, UK

## Abstract

Polyarteritis nodosa (PAN) is a multisystem, necrotising vasculitis of small- and medium-sized arteries with a predilection for the visceral vessels. Cutaneous PAN is a rare variant with symptomatic vasculitis limited to the skin, typically presenting as nodular lesions on the extremities with a propensity to ulcerate. We describe a rare case of histologically confirmed cutaneous PAN presenting in a 55-year-old Ghanaian woman with severe oropharyngeal ulceration. This was associated with dysphagia and significant weight loss. Oesophagoduodenoscopy showed that the ulceration extended throughout the oropharynx. Systemic polyarteritis nodosa was ruled out with magnetic resonance angiography. Our patient was treated successfully with corticosteroids and methotrexate. This case suggests that cutaneous PAN should be considered in the differential diagnosis of patients with oropharyngeal ulceration and that histological assessment is pivotal in establishing the diagnosis early in order to instigate appropriate therapy.

## 1. Introduction

Polyarteritis nodosa (PAN) is a multisystem, necrotising vasculitis of small- and medium-sized arteries with predilection for the visceral arteries. Cutaneous PAN is a rare variant with symptomatic vasculitis limited to skin, and at times, peripheral nerves [1]. The primary lesion is typically a subcutaneous nodule that affects the lower extremities, and these lesions often ulcerate [2]. Livedo reticularis has been reported in 50–80% of cases [3]. Commonly encountered symptoms include fever, malaise, myalgia, and arthralgia [4]. In this article, we present a rare case of histologically confirmed cutaneous PAN presenting with severe pharyngeal ulceration.

## 2. Case Presentation

A 55-year-old Ghanaian woman presented with a one-month history of mouth ulcers, painful swallowing, and fever. She had also developed cutaneous ulcers on the left lower leg, back, and scalp. She reported being nonspecifically unwell for 1 year, with fatigue, poor appetite, and 8 kg weight loss over three months. She was not taking any regular or new medications prior to this illness and had a background of asthma and asymptomatic mild-moderate mitral regurgitation. She did not consume alcohol or recreational drugs and smoked 10 cigarettes per day. She was born in Ghana and came to the UK aged 11. There was no travel history for 3 years prior to admission and no recent new sexual partners.

**Figure 1:**
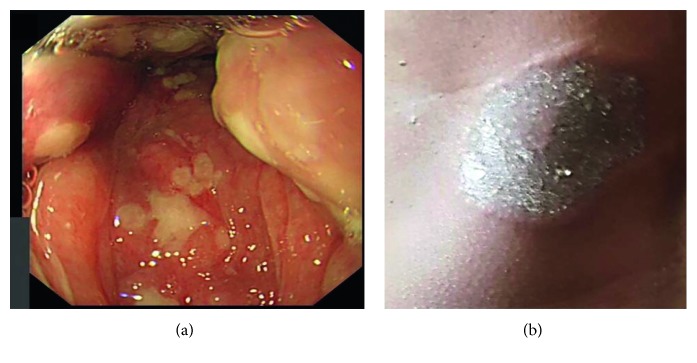
(a) Oesophagogastroduodenoscopy demonstrating extensive pharyngeal ulceration. (b) Ulcerating plaque (10 cm diameter) over the left central back; photograph taken by the patient's family member prior to admission. Reproduced with patient consent.

On admission, she was febrile at 38.2°C. Several painful mouth ulcers were seen, affecting the hard palate and the oropharynx (Figure 1(a)). No other mucous membrane involvement was present and specifically no genital ulcers. There was a 5 cm diameter circular pigmented nodule on the left calf, with central necrotic ulceration. She had a 10 cm diameter inflammatory well-demarcated plaque over the left central back (Figure 1(b)) and smaller ulcers over the scalp, all less than 1 cm diameter.

Laboratory tests revealed mild anaemia with a haemoglobin of 119 g/L, platelet count 227 × 10^9^/L, white cell count 9.0 × 10^9^/L, albumin 33 g/L (normal 35–55), an elevated erythrocyte sedimentation rate (ESR) of 69 mm/hr, and C-reactive protein (CRP) 73 mg/L (normal < 5). The ANCA test was negative, complement component C4 was elevated, and the rest of the autoimmune screen was negative. Blood and urine cultures and a scalp ulcer and throat swab revealed no bacterial growth. Viral throat swab was negative. Coeliac serology was also negative. Upper gastrointestinal endoscopy revealed marked oropharyngeal ulceration without contact bleeding (Figure 1(a)). A CT scan of the chest, abdomen, and pelvis revealed no evidence of malignancy, while an MR angiogram of the aorta revealed no evidence to suggest systemic PAN.

**Figure 2:**
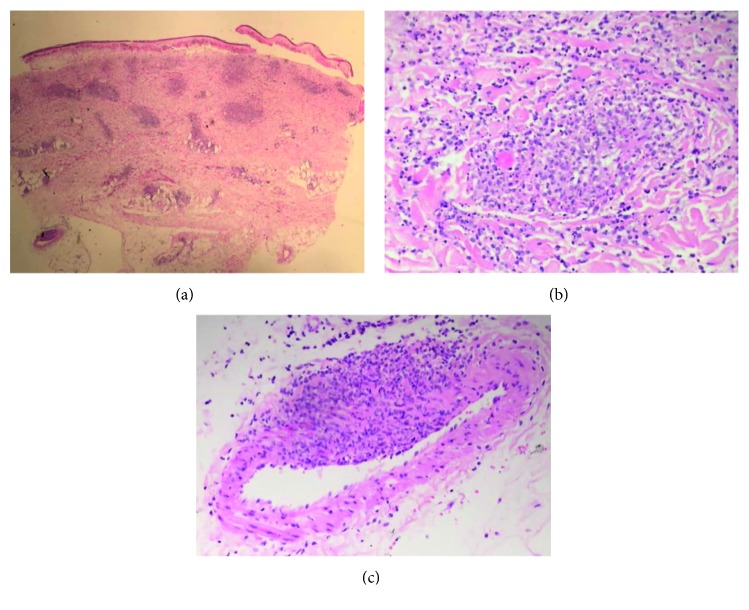
(a) H and E stained skin punch biopsy (x20 magnification) showing loss of the epidermis with an inflammatory infiltrate in the dermis composed of neutrophils and histiocytes, the latter coalescing into (b) poorly formed granulomas (x100 magnification). Abundant cytoclastic debris is present. (c) In areas the inflammation appears centred on blood vessels associated with fibrinoid necrosis (x200 magnification). The findings indicate a necrotising vasculitis with granulomatous features.

Two 4 mm punch biopsies were taken from the scalp lesions along with an 8 mm punch biopsy from the left lower leg nodule. These underwent haemotoxylin and eosin (H and E) and direct immunofluorescence staining. Immunohistochemistry from both locations revealed loss of epidermis with necrosis and a brisk inflammatory infiltrate in the dermis composed of neutrophils and histiocytes, the latter coalescing into poorly formed granulomas (Figures 2(a) and 2(b)). Abundant cytoclastic debris was present. In areas, the inflammatory cells were clearly centred around and obscuring blood vessels, which showed foci of fibrinoid necrosis (Figure 2(c)). The process involved small dermal blood vessels and larger muscular vessels deep in the biopsy. PAS (Periodic acid-Schiff) and ZN (Ziehl–Neelsen) stains were negative. Direct immunofluorescence was negative. The overall impression was of a necrotising vasculitis with granulomatous features, findings consistent with a diagnosis of cutaneous PAN.

Data are limited on the treatment used in cutaneous PAN. However, due to the severity of her presentation and oropharyngeal involvement precluding her from a safe swallow, systemic treatment was opted for in preference to topical therapy. The patient was initially prescribed intravenous hydrocortisone and subsequently oral prednisolone 40 mg and methotrexate 10 mg weekly. The latter was uptitrated to 15 mg weekly prior to discharge, and the prednisolone was slowly weaned. Once treatment was established, no further lesions developed and the existing oropharyngeal and cutaneous ulcers began to heal. After 23 days, when able to maintain good oral intake, the patient was discharged.

At clinic follow-up six weeks later, all the oral ulcers and the lesions on the leg, back, and scalp had healed, and no new lesions were seen. The patient reported persistent fatigue and muscle weakness, and there was evidence of mild proximal weakness in the hip flexors. Acute phase markers and serum albumin were within normal range. The methotrexate dose was increased from 15 mg to 17.5 mg weekly and the prednisolone dose was progressively weaned. At subsequent follow-up after 6 months, the patient was well with no active skin lesions. She continued methotrexate 20 mg weekly and prednisolone 5 mg daily. At 12-month follow-up, she remained in remission with CRP 2.0 g/L and ESR 24 mm/hr.

## 3. Discussion

The concept of a benign, limited cutaneous form of PAN has been debated since its first description in 1931 by Lindberg [5]. The precise pathogenesis of the condition is poorly understood. Cutaneous PAN presenting with typical nodular lesions on the extremities with a propensity to ulcerate is well recognised [2]. However, a presentation with severe mucocutaneous pharyngeal ulceration leading to significant weight loss has not been documented. Our case suggests that cutaneous PAN should be included in the differential diagnosis of oropharyngeal ulceration.

A diagnosis of cutaneous PAN requires exclusion of systemic PAN. This was confirmed in our patient by MR angiography. Although a diagnosis of systemic PAN can be supported by the aid of classification criteria, these do not include the cutaneous form of the disease [6]. In addition, whereas some have argued that cutaneous PAN can progress to the systemic form, Nakamura et al. concluded that although true cutaneous PAN may be associated with myalgia or neuropathy, this is limited to the same area as the skin lesions, and does not progress to the systemic form [7]. Moreland and Ball also discuss the differences between the two conditions, noting a 1 : 1 male to female ratio in cutaneous PAN and a significant male predominance in systemic PAN [8]. Unlike systemic PAN, the cutaneous form typically has a benign course with a good long-term prognosis and virtually no reports of mortality [2].

The subcutaneous tender, erythematous nodules (usually 0.5–3 cm in diameter) may disappear spontaneously or undergo ulceration. Other findings include petechiae, purpura, cutaneous necrosis, and autoamputations. Extracutaneous manifestations are not uncommon and include constitutional symptoms (commonly fever and malaise), arthralgia, myalgia, and peripheral neuropathy (mononeuropathy and mononeuritis multiplex) [1, 7].

Our case presented with pure cutaneous manifestations, and unusually oropharyngeal ulceration, without systemic involvement. Differential diagnoses for a blistering skin eruption and mucocutaneous ulceration were considered and excluded, including infection, underlying inflammatory bowel disease, dermatitis herpetiformis, Behçets syndrome, and drug reaction [9]. Oral ulceration is a very rare complication of polyarteritis nodosa and has been reported on occasion, including a study from the French vasculitis group [10]. There are no specific serological tests for cutaneous PAN, and diagnosis requires histopathologic confirmation [11]. Classically, skin biopsy reveals a necrotising vasculitis of the small arteries from the dermal-subcutaneous border to subcutaneous tissue, with infiltration of inflammatory cells into the media and intima together with fibrinoid degeneration [11]. Chen proposed a classification of cutaneous PAN into three stages: acute, repair, and scarring stages [12].

EULAR guidelines recommend the use of high-dose corticosteroids together with cyclophosphamide for remission induction in systemic PAN, followed by steroid-sparing agents including methotrexate, azathioprine, or leflunomide [13]. No specific guidelines for the management of cutaneous PAN have been developed. As cutaneous PAN is classically an ANCA-negative, nonorgan-threatening vasculitis, we adapted EULAR recommendations for the management of limited ANCA-associated vasculitis and prescribed corticosteroids and methotrexate [13]. This combination has proved effective for both cutaneous and oropharyngeal lesions to date, and use is supported by two previous reports of cutaneous PAN [14, 15].
